# Surgical margins and survival after head and neck cancer surgery

**DOI:** 10.1186/1472-6815-6-2

**Published:** 2006-02-17

**Authors:** Reina Haque, Richard Contreras, Michael P McNicoll, Evelyn C Eckberg, Diana B Petitti

**Affiliations:** 1 Department of Research & Evaluation, Kaiser Permanente Southern California, Pasadena, USA; 2 Head & Neck Surgical Oncology, Kaiser Permanente Los Angeles Medical Center Los Angeles, USA; 3 Department of Clinical Services, Southern California Permanente Medical Group Pasadena, USA

## Abstract

**Background:**

Mixed results exist as to whether positive surgical margins impact survival. The aim of this study was to determine whether positive surgical margins are indeed associated with decreased survival in patients with primary head and neck cancer.

**Methods:**

We conducted a retrospective cohort study of 261 cases diagnosed with cancer of the larynx or tongue between 1995 and 1999. Cases were followed through December 31, 2002. Survival curves by margin status were generated by Kaplan-Meier methods. Categorical data were evaluated with odds ratios (OR).

**Results:**

All-cause mortality was markedly higher in cases with positive margins as compared with those with negative margins (54% versus 29%, *P *= 0.005). This pattern also appeared after adjusting for age and sex (OR = 2.97, 95% CI: 1.29 – 6.84).

**Conclusion:**

Our findings suggest that positive surgical margin status is associated with increased mortality. This association also generally persists after adjustment for tumor size, stage, and adjuvant therapy.

## Background

Approximately 37,000 men and women in the U.S. were diagnosed with head and neck cancers in 2003 [1]. In particular, individuals with tongue and larynx cancer accounted for 45% of the head and neck cancer cases diagnosed. Such cancers are more frequently diagnosed among men and in people over age 50. These cancers are highly fatal, and mortality rates have not decreased significantly over time [1]. Based on recent national U.S. statistics, the five-year survival is 64% for larynx cancer and 56% for cancer of the oral cavity [1,2]. The treatment plan for an individual patient depends on a number of factors, including the exact location of the tumor, the stage of the cancer, and the person's age and general health status [3]. Patients with head and neck cancer are frequently treated with surgery, with removal of the cancer including some of the healthy tissue around it. Surgery may be followed by radiation treatment. But, the side effects of aggressive surgery and radiation can be devastating. Severe consequences may include permanent loss of voice, swallowing and speech problems, tongue and neck deformity and scar, and paralysis of cranial nerves. Significant expertise is needed to preserve healthy tissue to the extent possible to maintain these important functions while ensuring that the cancer is removed.

Although obtaining negative surgical margins (i.e., cancer was entirely removed) is the goal of the head and neck surgeon, achieving this may be impossible because of functional consequences. Thus, some patients are left with positive surgical margins (i.e., those in which residual cancer cells remain) to preserve vital organs like the carotid artery. Whether positive surgical margins impact survival remains equivocal [4]. Positive surgical margins are reported to be negatively associated with survival in many, although not in all of the published studies [5,6]. Some studies did not find an association between positive surgical margins and an increased risk of mortality; however, many of these studies included a small number of patients, or the possibility existed that there was no increase in mortality because patients with positive surgical margins received adjuvant radiation therapy [7,8]. In addition, many of the prior studies did not attempt to exclude cases with a known history of cancer, which may impact survival. The objective of this study was to determine whether positive surgical margins are indeed associated with lower survival.

## Methods

### Design and setting

The study was a retrospective cohort of larynx and tongue cancer cases diagnosed at Kaiser Permanente Southern California (KPSC), a health maintenance organization of 11 medical centers with over three million members. The study was reviewed and approved by the KPSC Institutional Review Board.

### Study patients

Study patients were identified through the health plan's cancer registry. The KPSC cancer registry is a population-based registry that reports to the American College of Surgeons' National Cancer Data Base and National Cancer Institute's Surveillance Epidemiology and End Results Program. Primary tongue and larynx cancer cases included patients who were diagnosed between January 1, 1995 and December 31, 1999 who underwent surgical resection as the primary course of treatment, and were active health plan members of KPSC on the date of surgery (International Classification of Disease for Oncology [ICD-O] Version 2 codes C01.9, C02.0–C02.4, C02.8, C02.9, C32.0–C32.3, C32.8, C32.9). To minimize the possibility of having biased survival times, we excluded patients with a known history of cancer.

Cases with negative margins included patients with no involvement of margins (i.e., reported free by the pathologist). Cases with positive margins were defined to be those in whom residual cancer cells were found in the surgical margin when tissue sections were examined with a microscope (microscopically positive) or visible to the unaided eye (grossly positive). These categories describe the surgical margins status after resection of the primary tumor as recorded by the pathologist.

### Data collection

Variables abstracted from the cancer registry included age at diagnosis, sex, ethnicity, ICD-O (International Classification of Diseases, Oncology) diagnosis, diagnosis date, TNM stage (tumor, node, metastases), surgery date, surgical margin status, radiation therapy, vital status, and cause of death.

Vital status was ascertained by using a combination of research and automated databases. Deaths were identified through the California's master file of death certificates, inpatient hospital files, the cancer registry, and the National Death Index. Because cause of death information was missing for over 90% of the patients in these automated databases, we used all-cause mortality as the endpoint.

We classified patients as being alive at end of study if they had any one of the following after December 31, 2002: 1) an outpatient doctor visit; 2) an inpatient hospitalization; or 3) received a pharmacy prescription. Because these patients have a highly fatal cancer requiring medical care, they would appear in one of these databases.

### Statistical analysis

The statistical significance of differences for categorical data was evaluated using chi-square or Fisher's exact test. Odds ratios (OR) were used to estimate the size of associations; ORs were adjusted for age and sex, and stratified by tumor characteristics and surgical treatment separately. For the survival analysis, patients were followed from the date of surgery to December 31, 2002. We examined overall survival defined as the interval between the date of surgery and December 31, 2002 or the date of death due to any cause, whichever occurred first. Survival time was censored if a patient lived past the end of follow-up. Survival curves by margin status were generated by Kaplan-Meier methods. Differences in survival between cases with positive and negative margins were evaluated using the log-rank test. All analyses were conducted with SAS Version 8 [9].

## Results

We identified 261 newly diagnosed cases through the health plan's cancer registry (51% with tongue cancer and 49% with larynx cancer). The number of cases diagnosed by year was relatively constant after 1995 (Table 1). More men were diagnosed with these cancers compared to females (71% versus 30%). The number of cases increased with age, with the majority of patients diagnosed between ages 60 and 69 (37%). Over 75% of the patients were white. Over 90% of the 261 total study patients had squamos cell carcinoma.

**Table 1:** Demographic characteristics of incident tongue and larynx cancer cases

	N (N = 261)	%
Year of diagnoses		
1995	48	18.4
1996	54	20.7
1997	58	22.2
1998	52	19.9
1999	49	18.8
		
Gender		
Male	185	70.9
Female	76	29.1
		
Age at diagnosis		
<40	11	4.2
40–49	33	12.6
50–59	66	25.3
60–69	96	36.8
70+	55	21.1
		
Ethnicity		
White	202	77.4
Black	24	9.2
Asian	16	6.1
Hispanic	16	6.1
Other/Unknown	3	1.2

A total of 233 patients had negative margins and 28 had positive margins (Table 2). Cases with negative margins were more likely to have tumors ≤2 cm in size, whereas those with positive margins had larger tumors (>2 cm). However, tumor size data was missing for over 30% of the cases. Although a 2 cm tumor for laryngeal cancer may be considered large, we found only two patients exist with laryngeal cancer who had <2 cm size margins and positive margins, thus making it impracticable to conduct analyses stratified by lower cut-off points. The majority of cases with negative margins presented with TNM stage 0 or I disease. In contrast, patients with positive margins were more likely to present with TNM stage IV disease and undergo adjuvant radiotherapy (*P *< 0.0001).

**Table 2:** Tumor characteristics by margin status in incident tongue and larynx cancer cases

	Negative Margins (N = 233)		Positive Margins (N = 28)		P*
	N	%	N	%	
Tongue					
Tumor size (cm)					
≤ 2	60	49.5	6	46.2	
>2 cm	34	28.2	5	38.5	
Missing	27	22.3	2	15.4	
					
TNM Stage					
0 – I	66	54.5	2	15.4	
II	22	18.2	2	15.4	
III	17	14.1	2	15.4	
IV	14	11.6	7	53.8	
Missing	2	1.7	0	--	
					
Larynx					
Tumor size (cm)					
≤ 2	23	20.5	2	13.3	
>2 cm	42	37.5	7	46.7	
Missing	47	42.0	6	40.0	
					
TNM Stage					
0 – I	44	39.2	3	20.0	
II	12	10.7	1	6.7	
III	29	25.9	0	--	
IV	25	22.3	11	73.3	
Missing	2	1.8	0	--	
					
Overall					
Primary site					
Tongue	121	51.9	13	46.4	
Larynx	112	48.1	15	53.6	
					
Tumor size (cm)					
≤ 2	83	35.6	8	28.5	0.575
>2 cm	76	32.6	12	42.8	
Missing	74	31.7	8	28.5	
					
TNM Stage					
0 – I	110	47.2	5	17.8	<0.0001
II	34	14.6	3	10.7	
III	46	19.7	2	0.7	
IV	39	16.7	18	64.2	
Missing	4	0.2	0	0	

*P value based on chi-square distribution. Not calculated for the individual primary sites due to small cell size.

Among cases with large tumors (>2 cm), mortality was dramatically greater in cases with positive margins as compared to those with negative margins (OR, 2.47, 95% CI: 0.69–8.91, Table 3). A similar association was not seen among those with smaller tumors (<2 cm); however, the number of individuals with positive margins was too small to draw an inference. We also examined the association by TNM stage. Because of the small number of patients with stage 0 disease, we combined this category with stages I to III in the analyses to allow for the calculation of the ORs. Mortality was over three-fold greater in cases with positive margins among those diagnosed with stage 0 to III disease (OR = 3.32, 95% CI: 0.92 – 11.99). There was no difference in mortality by surgical margins status when examining cases diagnosed with stage IV disease (OR = 1.07, 95% CI: 0.35 – 3.29) but the confidence interval was wide. Adjustment for age and sex did not affect the associations.

**Table 3:** Association between surgical margins and mortality by tumor characteristics

Therapy	Died (N)	Alive (N)	Odds Ratio (95% CI)	Age-sex adjusted OR (95% CI)
Tumor size				
≤ 2 cm				
Positive	7	1	0.45 (0.05 – 3.89)	0.41 (0.04 – 4.05)
Negative	63	20		
> 2 cm				
Positive	4	8	2.47 (0.69 – 8.91)	2.37 (0.58 – 9.75)
Negative	42	34		
				
TNM Stage				
0 – III				
Positive	5	5	3.32 (0.92 – 11.99)	3.34 (0.86 – 13.07)
Negative	146	44		
IV				
Positive	8	10	1.07 (0.35 – 3.29)	0.95 (0.26 – 3.51)
Negative	18	21		

Overall mortality was nearly three-fold greater in cases with positive margins as compared to those with negative margins (age-sex adjusted, OR = 2.97, 95% CI: 1.29 – 6.84, Table 4). Similar associations were seen among patients who underwent surgery only (154 cases) and among those treated with adjuvant radiotherapy (107 cases), although confidence intervals were again wide.

**Table 4:** Association between surgical margins and mortality by surgical treatment in cases with tongue or larynx cancer

Therapy	Died (N)	Alive (N)	Mortality (%)	Odds Ratio (95% CI)	Age-sex adjusted OR (95% CI)
All cases					
Positive	15	13	53.6	2.85 (1.19 – 6.89)	2.97 (1.29 – 6.84)
Negative	67	166	28.8		
					
Surgery only					
Positive	3	4	42.9	2.38 (0.33 – 14.85)	2.20 (0.42 – 11.55)
Negative	35	112	23.8		
					
Surgery + adjuvant radiotherapy					
Positive	12	9	57.1	2.23 (0.77 – 6.74)	1.98 (0.68 – 5.84)
Negative	32	54	37.2		

**Figure 1:**
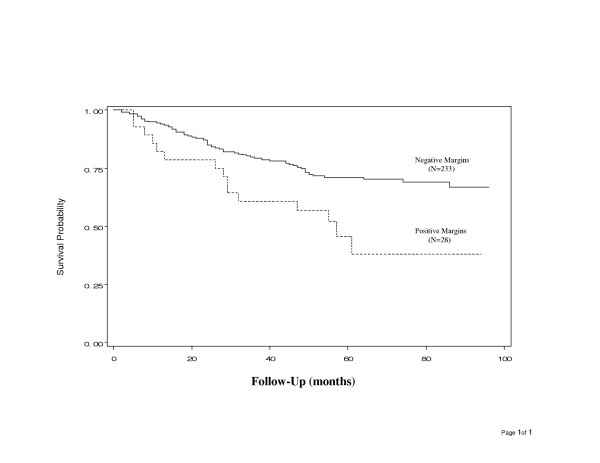
Comparison of overall survival in patients with cancer of tongue or larynx by surgical margin status.

In the survival analysis, cases were followed a maximum of 8 years (range of 2.5 to 96 months). We observed 82 deaths during the study period. The overall survival probability was 31%. All-cause mortality was markedly higher in cases with positive margins as compared with those with negative margins (54% versus 29%, *P *= 0.005, Table 4 and Figure 1).

Cause of death was not available in the various electronic files. We found cause of death for only 25 individuals. Of these 25 patients, 15 (60%) died from a malignant neoplasm of the head or neck; 5 (20%) due to cardiovascular disease; and 5 (20%) died due to pneumonia or other causes.

## Discussion

We examined the association between surgical margin status and survival using a cohort of 261 patients diagnosed from 1995 to 1999, with some patients followed five or more years. All-cause mortality was markedly higher in cases with positive margins as compared with those with negative margins (54% versus 29%) during the eight year study period. The age and sex adjusted OR suggests that positive surgical margin status is associated with nearly a three-fold increased risk of mortality (2.97, 95% CI: 1.29 – 6.84). This association appears to generally persist even after adjustment type of treatment (surgery alone or with adjuvant radiation therapy), among cases with large tumors (>2 cm), and those with TNM stage 0 to III disease at diagnosis; however, these analyses were based on small numbers of cases. These findings are consistent with the few reports that specifically examined the effect of surgical margins on survival of head and neck cancer [4,5,10,11]. Other studies did not find a clear association between positive surgical margins and an increased risk of death, possibly due to even smaller sample sizes to detect a difference (e.g. [7]), or did not specifically examine the effect of margins on survival.

Use of all-cause mortality is a potential limitation. We examined all cause mortality because cause of death information was available for only 25 cases who died. Using all-cause mortality would underestimate mortality due to cancer, but it is unlikely to lead to bias in estimation of the relationship of margins with survival since death due to causes other than cancer is unlikely to be related to margin status. In addition, we did not have information on chemotherapy in patients who might have experienced subsequent metastases. Indication for adjuvant therapy such as chemotherapy and radiotherapy would have entailed patient chart review which was beyond the scope of this study. Another potential limitation is was the lack of electronic data on the sub-anatomic sites of the tongue to examine the association between margin status and mortality. For example, it is more complicated to obtain negative margins for the base of tongue tumors. Although the present study includes one of the largest series investigated, the study only included 28 patients with positive surgical margins. Therefore, in the analyses we examined overall survival for the two primary sites combined that would be based on more stable estimates.

A major strength of the study is that it includes a large population-based cohort, which is more likely to be representative of all individuals who develop cancer of tongue and larynx than studies from academic specialty centers. Virtually all previous studies we identified were based on patients from academic specialty centers who might have been more likely to have more aggressive forms of the disease [4-12]. Another strength is that our study involved patients who were diagnosed over a five-year time frame, during which surgical and treatment modalities were unlikely to vary substantially. Some previous studies included patients who diagnosed over decades. Further, our study excluded patients with a known history of cancer. All patients receive all their health care within this integrated HMO; therefore, the study included virtually every case diagnosed and treated during the study period.

## Conclusion

Cancers of larynx and tongue are aggressive and prognosis is poor. Surgery has been the primary course of treatment for these diseases. Our results suggest that positive surgical margin status is associated with decreased survival. However, achieving negative margins can cause impairment in important functions such as chewing, swallowing and speech, and adversely affect quality of life. Measures of quality of life are not available in the health plans electronic databases, and ascertaining such information was beyond the scope of the present study. Detailed prospective data collection would be needed to understand the functional limitations experienced by patients with negative and positive margins. The appropriate balance between quality and quantity of life remains a difficult clinical decision.

## Competing interests

The author(s) declare that they have no competing interests.

## Authors' contributions

RH, RC and DP contributed substantially to the study design, data retrieval, statistical analysis, interpretation of the study results, and writing of the manuscript. MPN and EE contributed intellectually to the interpretation of the study results and drafting the manuscript and critically reviewing the final version. All authors have read and approved the final version.

## Pre-publication history

The pre-publication history for this paper can be accessed here:


